# University student mental health research: look back to move forward

**DOI:** 10.1192/bjo.2026.12024

**Published:** 2026-06-16

**Authors:** Anne Duffy, Rohan Borschmann, Aileen A. O’Brien, Lucy J. Robinson, Susan M. Sawyer, Edward A. Selby, Edward R. Watkins, Kenneth R. Kaufman

**Affiliations:** Department of Psychiatry, Queen’s University, Ontario, Canada; Health Service and Population Research Department, Institute of Psychiatry, Psychology and Neuroscience, King’s College London, UK; Centre for Adolescent Health, Murdoch Children’s Research Institute, Melbourne, Australia; Department of Psychiatry, University of Oxford, UK; City St George’s, University of London, UK; School of Psychology, Newcastle University, UK; Centre for Adolescent Health, Murdoch Children’s Research Institute and Royal Children’s Hospital, Melbourne, Australia; Department of Paediatrics, The University of Melbourne, Australia; Department of Psychology, School of Arts and Sciences, Rutgers, The State University of New Jersey, New Jersey, USA; School of Psychology, University of Exeter, UK; Departments of Psychiatry, Neurology, and Anaethesiology, Robert Wood Johnson Medical School, Rutgers University, New Jersey, USA; Department of Psychological Medicine, Institute of Psychiatry, Psychology and Neuroscience, King’s College London, UK

**Keywords:** Depressive disorders, epidemiology, longitudinal data, suicide, university student mental health

## Abstract

To celebrate the 10th anniversary of *BJPsych Open*, this Editorial highlights papers published in *BJPsych Open* over the past decade that have focused on university student mental health. Common mental disorders are increasing in young people and those going on to higher education make up an important and sizeable sector of this population. At the same time, success in university studies is a major determinant of individual and societal health and prosperity. As a field of inquiry, university student mental health research gained momentum through the COVID-19 pandemic and associated campus closures, pivot to remote learning and social restrictions. Although research describing student well-being and mental health burden align globally, not enough is known about determinants that inform sustainable and scalable prevention and early intervention. Furthermore, research evidence should inform university policies, practices and benchmarks to ensure responsive and effective student well-being and mental health support that underpins academic and life success.

**Figure:**
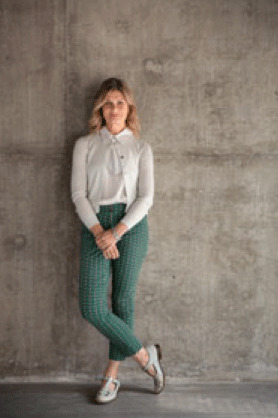

The prevalence of common mental disorders and self-harm have significantly increased over the past two decades in young people aged 16–25 years, a critical period in psychosocial and brain development that coincides with entry to higher education.^1^ Over this same period, access to university education has increased, leading to more enrolments of students from diverse and historically disadvantaged backgrounds who may be more vulnerable to mental health challenges and disorders.^2^ Although the number of incoming university students declaring a disability on the basis of a pre-existing mental disorder has dramatically increased, it is thought to represent only a fraction of those affected.^3^ In one study of college students from eight countries, depression and anxiety were estimated to affect a quarter and a third of undergraduate students, respectively, whereas lifetime self-harm was reported by about a fifth of students surveyed.^4^ Notably, the majority of students who might benefit from mental health support do not seek treatment.^4^ Untreated mental disorders among students often persist and are associated with worsening severity, reduced academic performance, increased attrition and increased risk of suicide.^1^ As success in higher education is a robust determinant of individual and societal health, university student mental health is a public health concern of global significance.^5^

The increase in scope and complexity of student mental health support need has strained university resources and, together with highly publicised student tragedies, have pressured universities to review their welfare services and reflect on their role in the provision of student mental health support.^6^ Position papers on the topic highlight the need for a sustainable, rationalised, evidence-informed framework of integrated mental health promotion and early intervention with embedded metrics fostering continual improvement and proactive (rather than reactive) development.^6^ The *BJPsych Open* has published several research articles focusing on university student mental health, and it is fitting as part of the 10th anniversary to look back on some of this work to inform future directions in research, practice and policy moving forward.

Student self-harm and suicide risk are understandably a major concern. Although suicide rates are lower than in the general population of the same age, rates in higher education students in England and Wales have increased since 2010.^7^ In *BJPsych Open*, a cross-sectional survey of Norwegian higher education students reported lifetime rates of suicidal thoughts and attempts at 21 and 4.2%, respectively, associated with being single, living alone, low annual income and immigrant student status.^8^ Other reported risk factors in the wider literature include substance misuse, academic problems and examination-related stress.

The onset of the COVID-19 pandemic and associated social restrictions, including the shift to online learning, prompted a number of research studies that explored the impact on student mental health. In *BJPsych Open*, Tang et al^9^ reported comparable rates of moderate to severe symptom levels of anxiety and depression (around 40%), insomnia (25%), substance misuse (31%) and suicidality (31%) in university students from the UK compared with age-matched controls, following the first COVID-19 lockdown from July to September 2020. Higher reported symptom levels were associated with younger age, pre-existing mental health conditions, lower income and sleep problems, as well as with male gender for substance misuse and other gender (non-binary) for suicidality. Six months later, there was evidence of a reduction in levels of anxiety and depressive symptoms, but no significant differences in insomnia, substance use or suicidality. These findings align with those globally of an increase in anxiety and depression coinciding with the peak of the pandemic, with a return toward pre-pandemic levels after the loosening of restrictions and the return to in-person learning.

Relatively few longitudinal studies have assessed university student mental health prior to, during and following the resolution of the COVID-19 pandemic. *BJPsych Open* published findings from a longitudinal cohort study of over 9500 Canadian undergraduates (spanning 2018 to 2022), which provided evidence of increased rates of probable insomnia up to the peak of the pandemic, with improvement in sleep quality after the pandemic.^10^ Students with poor sleep quality reported daytime problems with mood, energy, relationships and concentration. Similarly, Li et al^11^ found evidence of an increase in reported symptoms of depression, anxiety and stress in a cohort of Chinese students assessed at multiple time points from the start and over 28 months of the pandemic lockdown (2019–2022). In line with the broader literature linking social support and resilience, students locked down at university reported more mental distress compared with being locked down at home or not under any restrictions.

Other studies have focused on risk factors associated with common mental disorders in university students. A cross-sectional study of undergraduate students in Ireland published in *BJPsych Open* found evidence of an association between childhood adversity and suicidal ideation in students, and an indirect association through thwarted belongingness and perceived burdensomeness.^12^ Jarvers et al^13^ explored the relationship between non-suicidal self-injury and childhood trauma with emotional reactivity (i.e. sensitivity), aggression and depressive symptoms, highlighting the importance of lifetime trauma in contributing to emotional and behavioural problems in university students. A longitudinal study of Canadian undergraduate students found that clinically significant levels of anxiety and depressive symptoms, along with the modifiable risk factors of insomnia, binge drinking, cannabis use, social support, self-esteem and exercise at entry to university, predicted mental health and academic performance at the end of the first year.^14^ These findings align with the broader literature together with perfectionism, locus of control and repetitive negative thoughts as salient psychological risk factors and thereby prevention targets.

There has been debate about the role that universities should play when considering mental health support and how far the accepted duty of care extends, especially regarding students with diagnosed mental disorders. University charters and authoritative papers have suggested that universities should focus on a combination of universal well-being promotion and rationalised early intervention.^6^

Barriers to care and access inequities have been a particular focus of inquiry. In a qualitative case study of students with a history of self-harm attending an inner-city UK university, Tickell et al^15^ identified four themes relevant to help-seeking: (a) the transition to university is the time associated with the greatest challenges, (b) beliefs around having a valid mental health problem, (c) stigma fears around seeking support, and (d) the importance of flexible and facilitated treatment options and pathways. Bennett et al^16^ examined the impact of implementing a non-clinical well-being programme on university student help-seeking and mental health outcomes in the UK. Pre–post service introduction surveys provided evidence of a reduction in reported student anxiety and low well-being levels with no impact on depressive symptoms. Administrative service use data provided evidence of fewer student counselling referrals and improved student perception of access to support over the implementation period.

Despite a relative lack of rigorous study, peer support continues to gain wide acceptance among university leaders and peer counsellors alike as a sustainable and important offering in student welfare services.^17^ A systematic review of the literature pertaining to the effectiveness of peer support in higher education published in *BJPsych Open* found that firm conclusions were prevented because of heterogeneity of study measures and outcomes and poor-to-fair ratings of risk of bias in the 28 papers reviewed.^18^ Consistent with the wider literature, the authors concluded that there was ‘not a solid evidence base’ supporting the effectiveness of peer support for student mental health and well-being.

A selective look back at studies published in *BJPsych Open* over the past decade supports that anxiety, depression and self-harm are major issues in university students, and that their prevalence is increasing. Contributing factors appear to include academic stress, financial pressures, social isolation and sociopolitical uncertainties. Identifiable high-risk students include those from minoritised groups (i.e. based on sexuality, gender and/or ethnicity), with a history of childhood trauma and adversity, and a personal and/or family history of mental illness. Importantly, the transition to higher education represents a key window of opportunity for prevention and early intervention. In line with recommendations from studies published in *BJPsych Open* and the broader literature,^19,20^ mental health literacy that aims to reduce stigmatising attitudes and negative rumination; improve emotional self-awareness, compassion and allyship; and foster healthy coping and lifestyle choices seems a sensible, acceptable and effective universal prevention strategy. Further, psychological therapy to manage anxiety, depression and trauma-related symptoms also seems fundamental given the prevalence, persistence and impact of these conditions among students. Despite the popularity and appeal, there is little evidence of the effectiveness of peer support for symptomatic students, although it may possibly be an effective strategy to help combat stigma and build capacity and engagement in mental health promotion. Although universities should not attempt to duplicate specialised mental health services, they certainly have a central role to play in promoting well-being and advancing early detection and support of common mental health concerns in students under their care. Finally, facilitated pathways to local community-based specialised mental health services also seem key given concentrated academic terms and likelihood of studying away from home.

An important advance in student mental health research would be the adoption of a universal set of mental health screening measures with thresholds validated in student populations. Another potentially impactful research direction is the role that artificial intelligence and digital technology might play in enhancing early identification, facilitating access to indicated support, and building capacity for sustainable, rationalised and tailored care – following rigorous testing and provision of appropriate safeguards. Hybrid implementation and effectiveness studies of interventions would improve our understanding of important aspects of student and provider uptake to ensure successful scale and spread of resources and interventions. Additionally, a more in depth understanding of the determinants and mechanisms underlying student mental health concerns and a shift from reactive to proactive support development are warranted. Finally, the ready translation of evidence into benchmarks and standards across institutions and countries remains an important work in progress. Our youth represent the best investment in our collective future and higher education is an important determinant of a healthy society.
